# Complex hybrid weighted pruning method for accelerating convolutional neural networks

**DOI:** 10.1038/s41598-024-55942-5

**Published:** 2024-03-06

**Authors:** Xu Geng, Jinxiong Gao, Yonghui Zhang, Dingtan Xu

**Affiliations:** School of Information and Communication Engineering, Hainan University, Haikou, 570228 China

**Keywords:** Electrical and electronic engineering, Computational science, Computer science, Information technology

## Abstract

The increasing interest in filter pruning of convolutional neural networks stems from its inherent ability to effectively compress and accelerate these networks. Currently, filter pruning is mainly divided into two schools: norm-based and relation-based. These methods aim to selectively remove the least important filters according to predefined rules. However, the limitations of these methods lie in the inadequate consideration of filter diversity and the impact of batch normalization (BN) layers on the input of the next layer, which may lead to performance degradation. To address the above limitations of norm-based and similarity-based methods, this study conducts empirical analyses to reveal their drawbacks and subsequently introduces a groundbreaking complex hybrid weighted pruning method. By evaluating the correlations and norms between individual filters, as well as the parameters of the BN layer, our method effectively identifies and prunes the most redundant filters in a robust manner, thereby avoiding significant decreases in network performance. We conducted comprehensive and direct pruning experiments on different depths of ResNet using publicly available image classification datasets, ImageNet and CIFAR-10. The results demonstrate the significant efficacy of our approach. In particular, when applied to the ResNet-50 on the ImageNet dataset, achieves a significant reduction of 53.5% in floating-point operations, with a performance loss of only 0.6%.

## Introduction

In recent years, deep convolutional neural networks (CNNs) have achieved remarkable success across various research domains. Examples include rapid and accurate flood prediction models^1–3^, power system short-term voltage stability assessment with class imbalance^4^, global climate-driven factor forecasting^5^, image quality assessment^6^, soil erosion sensitivity assessment^7^, detection of false data injection attacks in smart grids^8^, as well as brain motor imagery classification in advanced bioengineering technologies^9–11^. These broad and significant applications prompt the development of more expansive and intricate architectures aimed at achieving enhanced performance^12,13^. Nevertheless, contemporary state-of-the-art CNNs often encompass a substantial number of weight parameters, consequently demanding significant memory and computational resources during inference. This characteristic poses challenges for their deployment on resource-constrained platforms, such as mobile devices. Even highly efficient neural network architectures, exemplified by residual connections, comprise millions of parameters and necessitate billions of floating-point operations (FLOPs)^14^. Hence, the quest for deep CNN models with a judicious balance between computational efficiency and precision underscores the importance of leveraging neural network pruning techniques.

The neural network pruning methods are categorized into structured pruning and unstructured pruning based on whether the pruning preserves the structured organization of filter parameters after the pruning process. Unstructured pruning^15–17^ involves the direct removal of weights with smaller L2 norms within the filters, leading to the creation of unstructured sparse neural networks. The core idea of^18^ is to iteratively compute and discard weights below a predefined threshold^19^. Formulates pruning as an optimization problem, where the goal is to search for weights that minimize the loss function while satisfying the pruning cost constraints. This irregular sparsity poses challenges in efficiently utilizing libraries such as the Basic Linear Algebra Subprograms. In contrast, structured pruning^20–25^ entails the direct removal of entire redundant filters, resulting in the formation of a regularly structured neural network model. Consequently, structured pruning contributes to enhanced network runtime performance. In the study conducted by^20^, the l1-norm criterion is employed to eliminate filters that are deemed insignificant. Similarly^21^, introduces the l2-norm criterion for filter selection and implements a technique called soft pruning on the selected filters. A pioneering approach proposed by^22^ promotes sparsity in the model through scaling parameters within the BN layers, thereby achieving highly effective pruning outcomes. To identify dispensable filters^23^, leverages spectral clustering techniques specifically tailored for filters. Another method, known as Filter Pruning via Geometric Median (FPGM)^24^, is employed to accurately trim redundant filters within the model. By introducing the Weighted Hybrid Criterion (WHC)^25^, a data-independent scheme robustly identifies the most redundant filters, taking into account factors such as filter size and linear correlations between filters, thus facilitating their targeted and precise pruning.

Structured pruning allows the use of computational acceleration libraries, while unstructured pruning, although capable of achieving the maximum pruning rate, cannot utilize computational acceleration libraries. Therefore, researchers prefer structured pruning. Regardless of the pruning strategy, an evaluation of the redundancy of filters must be conducted first, with evaluation criteria categorized into norm-based criteria and similarity-based criteria.

Filters with small norms are considered less crucial, while redundant filters exhibit similarity. However, these investigations predominantly focus on the convolutional layer alone. In contemporary neural networks, a BN layer is often introduced following the convolutional layer during training, aimed at stabilizing the input data for the subsequent convolutional layer^26^. This addition modifies the data distribution. The fundamental condition for the safe removal of a filter is the minimal impact on the subsequent convolutional layer. Notably, the data pipeline encompasses not only convolutional layers but also BN layers. Consequently, when undertaking pruning, it becomes imperative to account for the data transformation introduced by the BN layer.

**Figure 1:**
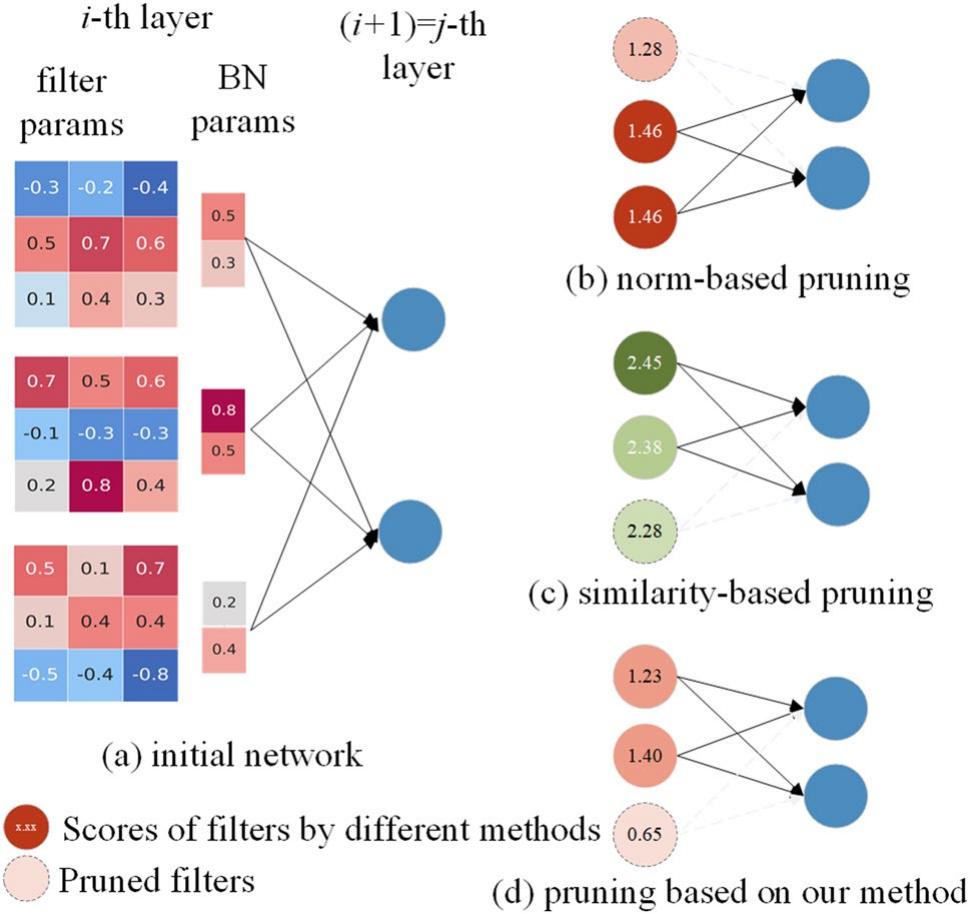
Score of filter (**a**) under different methods (**b**–**d**).

To mitigate the influence on the input of the subsequent convolutional layer, we present a novel method termed Complex Hybrid Weighted Pruning (CHWP). This approach accounts for both the convolutional layer and the BN layer, merging the norm-based and similarity-based criteria. In a detailed manner, we employ a weighted allocation approach to distribute the parameters of the BN layer among filters. This allocation is used to recalculate the norms of filters after applying the weighted distribution. Additionally, we utilize the norms of other filters as weights for the similarity of those filters. Subsequently, a score is computed for each filter, assigning higher scores to filters with larger norms and notable dissimilarities from other filters. Following this, filters with lower scores are identified and removed from consideration. It is noteworthy that CHWP differs from the criteria for filter selection based on norms and similarity. Even when the conditions set by these criteria are not met (Norm-based criteria require a large variance in the norms of these filters, while the similarity-based criterion performs poorly when all filters are dissimilar), its performance remains unaffected, as shown in Fig. 1. In figure 1, (a) is an example of a simple convolutional layer that does not fully satisfy the norm-based criterion and the similarity-based criterion. (b) and (c) are the scores for each filter in (a) based on the norm and based on the similarity criterion, respectively. The score distributions in (b) and (c) are quite concentrated with standard deviations of 0.08 and 0.06 respectively, which makes identifying redundant filters challenging. (d) applies our scoring method to score each filter in (a), with a standard deviation of 0.32, making the classification of whether a filter is redundant more obvious, thereby achieving robust performance.

The criteria based on norm and similarity are complementary. The norm-based criterion performs poorly when the norm distribution is concentrated, while the similarity-based criterion excels in such cases. However, the limitation of the similarity-based criterion is similar to that of the norm-based criterion; it is challenging to identify redundant filters when all filters are dissimilar. These two methods assess filter redundancy from different perspectives. Consequently, we combine these two criteria and propose the CHWP (Complex Hybrid Weighted Pruning) method. Following the principle of minimizing the impact on the input of the next layer, CHWP aims to better identify redundant filters with both a concentrated norm distribution and low similarity. We calculate scores for each filter using the CHWP method, considering filters with low scores as redundant. Extensive experiments on two benchmark datasets validate the effectiveness and efficiency of the proposed method.

## Methods

### Preliminaries

In this subsection, we introduce the symbols and notations used to describe neural networks. We assume a neural network with *L* convolutional layers and BN layers. We use $N_l$ and $N_{l+1}$ to denote the number of input and output channels of the *l*-th convolutional layer, and $F_{li}$ to denote the *i*-th filter of this layer, $F_{li}\in \mathbb {R}^{N_l\times K\times K}, 1\le l \le L, 1\le i \le N_{l+1}$, *K* denotes the size of the convolution kernel. $\gamma _{li}$ and $\beta _{li}$ represent the *i*-th parameter pair of the *l*-th BN layer.

### Analysis of norm-based and similarity-based criterion

**Figure 2:**
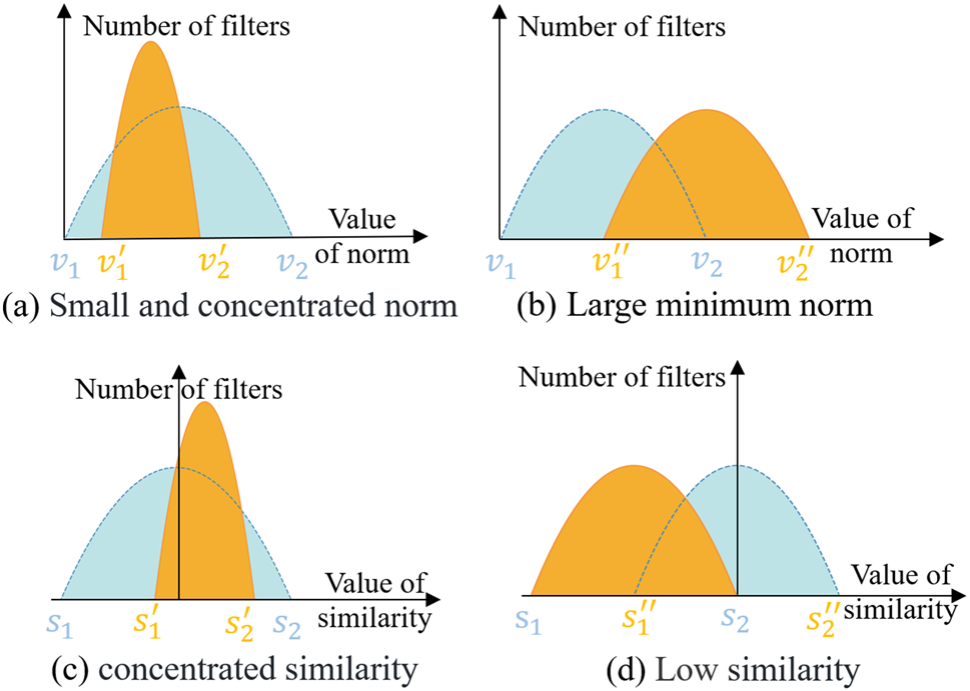
Ideal and reality based on norms and similarity criteria. The blue dashed curve represents the ideal distribution, while the orange solid curve represents the distribution that might occur in practical situations. *v* and *s* respectively denote the values of norm and similarity.

Several approaches mentioned earlier have demonstrated the utilization of norm-based and similarity-based criteria. However, in certain models, these criteria may not be well-suited, leading to unpredictable outcomes. This is illustrated in Fig. 2, where the blue dashed line and yellow solid line represent the ideal distribution and the actual distribution of filter norms or similarity, respectively.

As depicted in Fig. 2a, the deviation of the filter norm distribution may be too small, indicating that the norm values are highly concentrated within a narrow range. This makes it challenging to identify suitable thresholds for selecting filters to be pruned. In the case shown in Fig. 2b, where the smallest filter norm is relatively large, filters that are considered irrelevant by norm-based criteria may still have a significant impact on the network. This implies that pruning these filters could result in severe negative consequences. Similar to norm-based criteria, the distribution of filter similarity scores depicted in Fig. 2c exhibits excessive concentration, where the narrow range of scores makes it challenging to select an appropriate threshold for filter pruning. In Fig. 2d, the highest cosine similarity scores among the filters in the model remain notably low. In other words, the filters demonstrate significant dissimilarity. For example, similarity-based criteria would treat (0, 0.1) and (1, 0) as equally important. Under such circumstances, criteria based on similarity cannot effectively accomplish the intended purpose.

**Figure 3:**
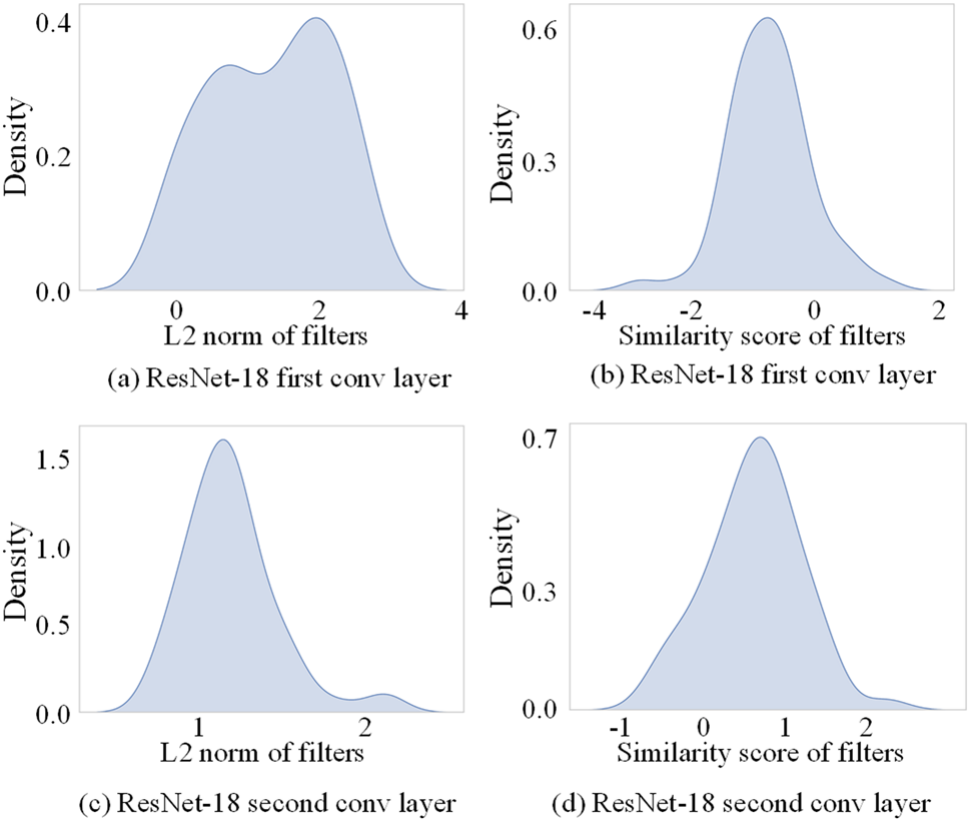
The distribution of filter norms and similarity scores for the first two convolutional layers of ResNet-18.

The statistical data obtained from ResNet-18 pre-trained on ImageNet^27^, presented in Fig. 3, substantiates the previous rule-based analysis. The norm or similarity distribution is plot in the kernel density estimation curve, a non-parametric technique for estimating the probability density of random variables.

In the case of the first convolutional layer in ResNet-18, as shown in Fig. 3a, a large number of filter norms are distributed near 0 to 3, which is close to a uniform distribution, making it suitable for norm-based criteria. Conversely, as illustrated in Fig. 3c, the norms in the second convolutional layer of ResNet-18 are concentrated in the range of 1 to 1.5, close to a normal distribution. Compared to the observed range of norms in the first layer, this distribution is noticeably narrower, making it challenging to set an appropriate threshold to distinguish the importance of filters.

For the first convolutional layer of ResNet-18, the scores based on similarity criteria, as shown in Fig. 3b, have the majority of filter norms falling within the interval [− 2, 2]. The dense distribution of filters presents a challenge in selecting the optimal threshold for differentiating critical filters. This is because similarity criteria consider filters with lower scores (greater dissimilarity) as more critical, but there are few filters in the low-score range of [− 4, − 2]. Regarding the second convolutional layer of ResNet-18, as depicted in Fig. 3d, the scores for these filters approximate an ideal distribution, making similarity-based criteria suitable.

By analyzing and comparing, it is determined that the first convolutional layer is more suited to norm-based criteria, while the second convolutional layer is better suited to similarity-based criteria. In practice, calculating scores for these filters based on both criteria and manually selecting the appropriate criterion can be time-consuming and labor-intensive. Therefore, this paper combines both methods using a weighted approach, eliminating the need for manual analysis and criterion selection.

### Complex hybrid weighted pruning

**Figure 4:**
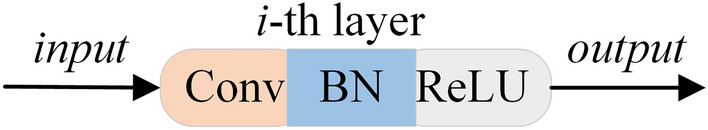
The operation that data flows from the current layer to the next layer.

Pruning aims to remove redundant filters that have the least impact on the next layer (convolutional or fully connected layer). The computation process from the current layer to the next layer is illustrated in Fig. 4, where data not only undergo convolutional operations but also pass through BN layers for scaling and shifting. When pruning redundant filters, corresponding BN layer parameters need to be removed as well. Therefore, pruning requires simultaneous consideration of both convolutional and BN layer parameters.

The computation of the BN layer is described by Eq. (1), where $\mu$ and $\sigma$ are the mean and variance of all feature maps in the *l*-th layer. $x_i$ represents the feature map output of the *i*-th channel in the convolutional layer, and $y_i$ is the corresponding output of the BN layer. $\varepsilon$ is a small positive constant added to prevent division by zero. $\gamma _i$ and $\beta _i$ are learnable parameters used for scaling and shifting the normalized values. They are trained through backpropagation to enable the network to adapt to the distribution of the data. These computations are independently performed for each feature channel. These computations demonstrate that the BN layer performs learnable scaling and shifting on the feature maps of the convolutional layer before they are input to the next convolutional layer. Therefore, we believe that when pruning, the learnable parameters of the BN layer should also be taken into consideration.

1 $$\begin{aligned} y_{i} = \gamma _{i} \frac{x_{i} - \mu }{\sqrt{\sigma ^2 + \varepsilon }} + \beta _{i} \end{aligned}$$

We propose a complex hybrid weighted pruning method to robustly prune redundant filters while minimizing their impact on subsequent network layers. In CWHP, there are two instances of weighting. The first instance involves weighting the filter norms using the parameters of the BN layer. The second instance involves weighting the dissimilarity using the filter norms. This method takes into account not only the norms and similarities of filters in the convolutional layers but also the parameters of the BN layers. The importance score calculation for the *i*-th filter $F_{li}$ in the *l*-th layer is as follows:

2 $$\begin{aligned} score_{li}=\psi _{(l,i)}\sum \limits _{j=1,j\ne i}^{N_{l+1}}\psi _{(l,j)}(1 - \left| \cos {\theta _{i,j}}\right| ), \end{aligned}$$

where

3 $$\begin{aligned} \psi _{(l,i)}= & {} \gamma _{li}\Vert F_{li}\Vert _2 + \alpha \beta _{li}, \end{aligned}$$

4 $$\begin{aligned} \cos \theta _{i,j}= & {} \frac{<F_{li},F_{lj}>}{\Vert F_{li}\Vert _2 \cdot \Vert F_{lj}\Vert _2}, \end{aligned}$$

and $\Vert F_{li}\Vert _{2}$ represents the *l*2 norm of the filter parameters $F_{li}$. In Eq. (2), the first part $\psi _{(l,i)}$ represents the norm-based significance of filter $F_{li}$ after being weighted by the parameters of the BN layer, while the remaining part(excluding $\psi _{(l,i)}$), indicates the cumulative dissimilarity between filter $F_{li}$ and other filters.

To justify the functioning of CHWP theoretically when applying CHWP in Eq. (2), we first discuss the $\psi _{(l,i)}$ component. Following the prevalent use of CNN-based design models, the forward computation process involves convolution operations followed by the BN layer. As shown in Eq. (3), Due to the scaling and shifting performed by the BN layer on the feature maps, we also apply corresponding scaling and shifting to the L2 norm of the feature maps, denoted as $\Vert F_{li}\Vert _2$. Here, $\alpha$ is a hyperparameter that balances the influence of $\gamma$ and $\beta$.

In Eq. (2), the dissimilarity metric is defined as $1 - \left| {\cos {\theta _{i,j}}} \right| \in [0,1]$, with $\psi$ as the weighting parameter. This metric effectively enhances the relationship between filter norms and dissimilarity, addressing the challenge of norm-based criteria losing effectiveness when norms are close. Additionally, unlike traditional Euclidean distance or angle-based distance^28^, CHWP select filters that are more orthogonal to other filters. This is because their projection lengths onto other filters are relatively short, making it advantageous for removing more redundant features.

In CHWP, we directly use filter norms and BN layer parameters as weights, effectively eliminating blind spots associated with norm-based and similarity-based criteria. When dealing with filters that exhibit minimal norm discrepancies, CHWP adeptly utilizes dissimilarity information to evaluate filters and identify those with the highest redundancy. When facing filters with relatively high angular similarity, it can select critical filters based on norm information. There is a scenario in which CHWP’s efficiency may decrease, which is when the scores computed by CHWP for various filters are close to each other. However, this situation implies the absence of redundancy, thereby negating the need to prune the corresponding model.

### Algorithm description

**Figure 5:**
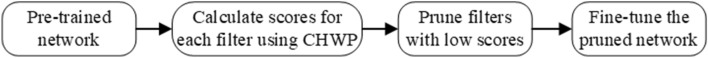
“Pretrain-prune-finetune” pipeline mechanism flow chart.

As described in Algorithm 1, we employ CHWP to execute filter pruning following the common “Pretrain-Prune-Finetune” pipeline mechanism (as shown in Fig. 5), whereby pruning is conducted at the different pruning rate for each layer. Although iterative mechanisms^29^, knowledge distillation^20,30^, sensitivity analysis for determining layered pruning rates , and certain fine-tuning techniques have been demonstrated to enhance the performance of pruned CNNs, we have refrained from utilizing these methods for the purpose of presentation and validation.

**Algorithm 1:**
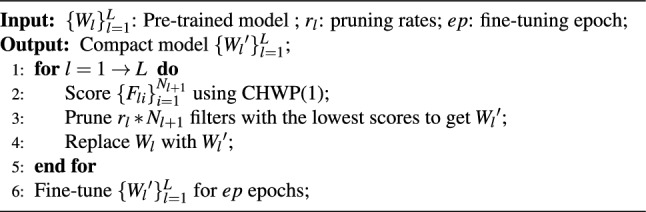
Algorithm Description of CHWP

### Ethical and informed consent for data used

The data used in this study were publicly available data sets on the Internet. No animals or humans were victims.

## Results and discussions

### Experimental settings

Following SFP and FPGM, we utilized several ResNet models of different depths for experiments conducted on both the CIFAR-10 (Canadian Institute for Advanced Research, 10 classes)^31^ and ImageNet datasets^27^. The reason we use these datasets and models is for ease of comparison with other pruning methods, as these datasets and models are widely adopted. We assess CHWP on various-depth ResNet models with pruning rates set at 40%, 50%, and 60% for those datasets.

The CIFAR-10 dataset is a subset of the Tiny Images dataset, comprising 60,000 $32 \times 32$ color images. Each image is assigned to one of the 10 mutually exclusive classes: airplane, automobile, bird, cat, deer, dog, frog, horse, ship, and truck. For each class, there are 6000 images in total, with 5,000 images designated for training and 1000 for testing. The relatively low resolution of the images, coupled with the small size of the objects within them, imposes higher performance requirements on algorithms being evaluated. The CIFAR-10 dataset is widely used in the development, testing, and comparison of various machine learning and deep learning models within the computer vision domain.

The ImageNet dataset is a large-scale visual recognition dataset containing over 1.2 million training images and 50K validation images spanning 1000 distinct classes. Each class represents a wide range of object categories, encompassing animals, and objects. This dataset is a fundamental resource for training and evaluating computer vision models, particularly those designed for image classification tasks. ImageNet has played a crucial role in advancing the field of deep learning, serving as the basis for the ImageNet Large Scale Visual Recognition Challenge (ILSVRC), which has been pivotal in benchmarking state-of-the-art image classification algorithms.

We conducted experiments using the Python programming language on the PyTorch deep learning platform. We maintained consistent experimental settings as outlined in the FPGM and WHC, which encompassed data augmentation strategies, pruning configurations, and fine-tuning. We use the accuracy of the unpruned pre-trained model as the baseline. Due to the fact that we pretrain the network in a different deep learning framework than a few other methods, there is a slight discrepancy (less than 0.5%) in our baseline compared to theirs. Therefore, our primary focus lies on examining the relationship between the reduction in FLOPs and the corresponding drop in accuracy. During the pruning phase, for a clearer comparison, we adopted the same pruning strategy as SFP and FPMG. This also implies that their reduction rates in FLOPs (Floating Point Operations) are identical. CHWP was compared against a selection of well-established methodologies, including data-independent norm-based PFEC^20^, SPF^21^, relation-based FPGM^24^, WHC^25^, ASPF^32^, as well as various data-dependent techniques such as HRank^33^, GAL^34^, LFPC^35^, CP^36^, NISP^37^, ThiNet^38^ and ABC^39^.

### Evaluation on CIFAR-10

In order to reduce experimental errors, we conducted three repeated experiments on the CIFAR-10 dataset, and the results were averaged. The results presented in Table 1 demonstrate the average accuracy achieved after fine-tuning. Table 1 clearly shows that the proposed CHWP method outperforms the several pruning methods that have been proposed in recent years. Specifically, in the case of ResNet-110, CHWP achieves a remarkable reduction in FLOPs by 65.8%, while maintaining minimal impact on average accuracy. In contrast, under the same experimental conditions, the rule-based SFP method experiences a notable decrease of 0.78% in accuracy. Furthermore, when compared to the pioneering WHC method, CHWP exhibits a competitive performance. These results suggest that CHWP, when applied at a moderate pruning ratio, effectively mitigates model overfitting and removes redundant filters without compromising overall model performance.

When compared to iterative ASFP, data-driven HRank, automl-based ABC, and LFPC, CHWP achieves a greater reduction in FLOPs in both ResNet-56 and ResNet-110. Remarkably, in terms of accuracy, CHWP surpasses LFPC by 0.42% and 0.75% for ResNet-56 and ResNet-110, respectively. This underscores CHWP’s effectiveness in identifying the most redundant filters and underscores the importance of considering BN layers during the pruning process. Furthermore, when compared to the aforementioned methods and at similar pruning rates, as the depth of the CNN increases, CHWP demonstrates a smaller decline in performance for the pruned models. This phenomenon can be attributed to the fact that deeper CNNs inherently contain more redundancy, which CHWP robustly eliminates without significantly compromising the CNN’s capacity.

In experiments on the CIFAR-10 dataset, it can be observed that as the depth of the network increases, the redundancy of CNN parameters gradually increases. These redundant parameters interfere with the decision-making of the CNN. For a ResNet with a depth of 20, when the FLOPs are reduced by 42.2%, the accuracy decreases by 0.16%. Interestingly, for a depth of 101, when the FLOPs decrease by 40.8%, the accuracy actually increases by 0.67%. As the pruning rate increases, the number of redundant parameters decreases. When FLOPs decrease by 65.8%, the accuracy increases by 0.16%. This result indicates that training larger CNN models on small datasets is prone to overfitting. Proper pruning can reduce computational load, alleviate overfitting, and maintain model performance.

**Table 1:** Pruning results on CIFAR-10. “$\downarrow$” means “drop”. In “Acc. $\downarrow$”, the smaller, the better; a negative drop means improvement. In “FLOPs $\downarrow$”, a larger number indicates that more FLOPs are reduced.

Depth	Method	Baseline acc. (%)	Pruned acc. (%)	Acc. $\downarrow$(%)	FLOPs $\downarrow$(%)
20	CHWP	92.20 (± 0.10)	92.04 (± 0.16)	0.16	42.2
	CHWP	92.20 (± 0.10)	91.42 (± 0.16)	0.78	54
32	CHWP	92.63 (± 0.60)	92.72 (± 0.06)	− 0.09	41.5
	CHWP	92.63 (± 0.60)	92.50 (± 0.09)	0.13	53.4
56	PFEC	93.03	93.06	− 0.02	27.6
	GAL	93.26	93.38	0.12	37.6
	SFP	93.57	93.78	− 0.21	41.4
	CHWP	93.59 (± 0.39)	93.96 (± 0.35)	− 0.37	41.4
	HRank	93.26	93.17	0.09	50
	SFP	93.59 (± 0.58)	93.35 (± 0.31)	0.24	52.6
	ASFP	93.59 (± 0.58)	93.12(± 0.20)	0.47	52.6
	FPGM	93.59 (± 0.58)	93.26 (± 0.03)	0.33	52.6
	CHWP	93.59 (± 0.58)	93.51 (± 0.11)	0.07	52.6
	LFPC	93.59 (± 0.58)	93.24 (± 0.17)	0.35	52.9
	ABC	93.26	93.23	0.03	54.1
	CHWP	93.59 (± 0.58)	93.67 (± 0.19)	− 0.08	54.8
	GAL	93.26	91.58	1.68	60.2
	CHWP	93.59 (± 0.58)	93.35 (± 0.08)	0.24	63.2
110	GAL	93.5	93.59	− 0.09	18.7
	PFEC	93.53	93.3	0.23	38.6
	SFP	93.68 (± 0.32)	93.86 (± 0.21)	− 0.18	40.8
	ASFP	93.68 (± 0.32)	93.37 (± 0.12)	0.31	40.8
	CHWP	93.68 (± 0.32)	94.35 (± 0.15)	− 0.67	40.8
	GAL	93.26	92.74	0.76	48.5
	SFP	93.68 (± 0.32)	92.90 (± 0.18)	0.78	52.3
	FPGM	93.68 (± 0.32)	93.74 (± 0.10)	− 0.06	52.3
	ASFP	93.68 (± 0.32)	93.10 (± 0.20)	− 0.39	52.3
	CHWP	93.68 (± 0.32)	94.09 (± 0.19)	− 0.41	52.3
	Hrank	93.5	93.36	0.14	58.2
	LFPC	93.68 (± 0.32)	93.07 (± 0.15)	0.61	60.3
	ABC	93.5	93.58	− 0.08	65
	WHC	93.68 (± 0.32)	93.82 (± 0.08)	− 0.14	65.8
	CHWP	93.68 (± 0.32)	93.84 (± 0.07)	− 0.16	65.8

### Evaluation on ImageNet

Alongside top-1 accuracy, we incorporate top-5 accuracy as a metric due to the ImageNet dataset’s extensive collection of images, many containing multiple objects. Each image is assigned only one true label. Given that the algorithm’s classification result may correspond to one of the objects in the image, which might not align with the provided true label, we deem the algorithm prediction correct if it predicts one of the top 5 objects, and one of them matches the ground truth.

Three experiments were conducted using the ImageNet dataset, and the results are comprehensively presented in Table 2. As expected, CHWP not only achieved the highest top-1 and top-5 accuracies, surpassing several state-of-the-art approaches, but also exhibited the least degradation in performance. Specifically, in the case of ResNet-50, CHWP effectively reduced FLOPs by over 40% while experiencing minimal compromises in both top-1 and top-5 accuracies. In contrast, the norm-based SFP method encountered a significant decline of 14% in top-1 accuracy, surpassing the 1% threshold observed in other methods.

For ResNet-50, with pruning rates set at 50%, our pruned model outperforms FPGM by 0.7% and 0.2% in Top-1 and Top-5 accuracy, respectively. Additionally, for the pruned pre-trained ResNet-101, CHWP reduces model FLOPs by 42.2%. Surprisingly, top-5 accuracy improves by 0.31%, and top-1 accuracy increases by 0.42%. At this point, FPGM experiences a performance decline of 0.02%, while WHC sees an improvement of 0.38%. Compared with norm-based and relation-based criteria, CHWP’s superior performance can be attributed to its synergistic utilization of both filter norm and similarity information, in conjunction with BN layer parameter pairs. This approach yields more robust and resilient results.

**Table 2:** Pruning results on ImageNet.“acc.” and“$\downarrow$” stand for “accuracy” and “drop”, respectively.

Depth	Method	Baseline top-1 acc. (%)	Pruned top-1 acc. (%)	Top-1 acc.$\downarrow$(%)	Baseline top-5 acc. (%)	Pruned top-5 acc. (%)	Top-5 acc.$\downarrow$(%)	FLOPs $\downarrow$(%)
18	SFP	70.23	60.79	9.44	89.51	83.11	6.4	41.8
	ASFP	70.23	68.02	2.21	89.51	88.19	1.32	41.8
	FPGM	70.28	68.41	1.87	89.63	88.48	1.15	41.8
	CHWP	69.76	68.68	1.08	89.08	88.82	0.26	41.8
34	PFEC	73.23	72.17	1.06	–	–	–	24.2
	ABC	73.28	70.98	2.3	91.45	90.05	1.4	41
	SFP	73.92	72.29	1.63	91.62	90.9	0.72	41.1
	ASFP	73.92	72.53	1.39	91.62	91.04	0.58	41.1
	FPGM	73.92	72.54	1.38	91.62	91.13	0.49	41.1
	CHWP	73.31	73.01	0.3	91.42	91.19	0.23	41.1
50	ThiNet	72.88	72.04	0.84	91.14	90.67	0.47	36.7
	SFP	76.15	62.14	14.01	92.87	84.6	8.27	41.8
	ASFP	76.15	75.53	0.62	92.87	92.73	0.14	41.8
	FPGM	76.15	75.59	0.56	92.87	92.63	0.24	42.2
	CHWP	76.13	76.12	0.01	92.86	92.86	0	42.2
	HRank	76.15	74.98	1.17	92.87	92.33	0.54	43.8
	GAL	76.15	71.95	4.2	92.87	90.94	1.93	43
	CFP	75.3	73.4	1.9	92.2	91.4	0.8	49.6
	CP	–	–	–	92.2	90.8	1.4	50
	FPGM	76.15	74.83	1.32	92.87	92.32	0.55	53.5
	CHWP	76.13	75.53	0.6	92.86	92.52	0.34	53.5
	GAL	76.15	71.8	4.35	92.87	90.82	2.05	55
	ABC	76.01	73.86	2.15	92.96	91.69	1.27	54.3
	LFPC	76.15	74.46	1.69	92.87	92.04	0.83	60.8
	CHWP	76.13	74.95	1.18	92.86	92.46	0.4	60.9
101	FPGM	77.37	77.35	0.02	93.56	93.55	− 0.01	42.2
	WHC	77.37	77.75	− 0.38	93.55	93.84	− 0.29	42.2
	CHWP	77.37	77.79	− 0.42	93.55	93.86	− 0.31	42.2
	ABC	77.38	75.82	1.56	93.59	92.74	0.85	59.8
	WHC	77.37	76.63	0.74	93.55	93.30	0.25	60.8
	CHWP	77.37	76.71	0.66	93.55	93.42	0.13	60.8

### Ablation study

To further validate the efficacy of CHWP, ablation experiments were conducted to gradually decouple CHWP into distinct sub-components, as depicted in Table 3. In order to facilitate a comprehensive comparison, the results of cosine criterion were incorporated. We performed three rounds of 40% filter pruning on ResNet-32 and ResNet-56, and reported the average decrease accuracy after fine-tuning. Compared to the cosine similarity criterion^40^, the dissimilarity metric (DM) exhibited lesser precision degradation. Taking into account the norms and dissimilarities, WHC based on a hybrid rule achieved favorable outcomes. In contrast to other methods presented in the table, CHWP yielded the most promising experimental results. In comparison to WHC, our devised CHWP demonstrated performance improvement in both ResNet-32 and ResNet-56, indicating the significance of employing a hybrid rule and considering the influence of BN layers. As the considered factors in the criteria become more comprehensive, the precision of removing redundant filters increases. The improvements in accuracy for HC, WHC, and CHWP (0.19% and 0.22%, respectively) demonstrate the equal significance of norm-based, relation-based criteria, and the introduction of BN layer.

**Table 3:** Decoupling results on ResNet-32 and ResNet-56 for CIFAR-10.“acc.” and“$\downarrow$” stand for“accuracy” and “drop”, respectively.

Depth & acc.	Criterion	acc. $\downarrow$(%)
32 92.63%	${\left\| {{F_{li}}} \right\| _2}$ (^21^)	0.42
	$\sum \nolimits _{j = 1}^{{N_{l + 1}}} {\cos {\theta _{i,j}}}$ (^40^)	0.78
	$\sum \nolimits _{j = 1}^{{N_{l + 1}}} {(1 - \left| {\cos {\theta _{i,j}}} \right| )}$ (DM)	0.56
	${\left\| {{F_{li}}} \right\| _2}\sum \nolimits _{j = 1}^{{N_{l + 1}}} {(1 - \left| {\cos {\theta _{i,j}}} \right| )}$ (HC)	0.32
	${\left\| {{F_{li}}} \right\| _2}\sum \nolimits _{j = 1}^{{N_{l + 1}}} {{{\left\| {{F_{lj}}} \right\| }_2}(1 - \left| {\cos {\theta _{i,j}}} \right| )}$ (WHC)	0.13
	$({\gamma _{lk}}{\left\| {{F_{li}}} \right\| _2} + \partial {\beta _{lk}})\sum \nolimits _{j = 1}^{{N_{l + 1}}} {({\gamma _{lk}}{{\left\| {{F_{lj}}} \right\| }_2} + \partial {\beta _{lk}})(1 - \left| {\cos {\theta _{i,j}}} \right| )}$ (CHWP)	− 0.09
56 93.59%	${\left\| {{F_{li}}} \right\| _2}$ (^21^)	0.20
	$\sum \nolimits _{j = 1}^{{N_{l + 1}}} {\cos {\theta _{i,j}}}$(^40^)	0.51
	$\sum \nolimits _{j = 1}^{{N_{l + 1}}} {(1 - \left| {\cos {\theta _{i,j}}} \right| )}$(DM)	0.37
	${\left\| {{F_{li}}} \right\| _2}\sum \nolimits _{j = 1}^{{N_{l + 1}}} {(1 - \left| {\cos {\theta _{i,j}}} \right| )}$(HC)	0.14
	${\left\| {{F_{li}}} \right\| _2}\sum \nolimits _{j = 1}^{{N_{l + 1}}} {{{\left\| {{F_{lj}}} \right\| }_2}(1 - \left| {\cos {\theta _{i,j}}} \right| )}$(WHC)	0.12
	$({\gamma _{lk}}{\left\| {{F_{li}}} \right\| _2} + \partial {\beta _{lk}})\sum \nolimits _{j = 1}^{{N_{l + 1}}} {({\gamma _{lk}}{{\left\| {{F_{lj}}} \right\| }_2} + \partial {\beta _{lk}})(1 - \left| \cos {\theta _{i,j}}\right| )}$ (CHWP)	− 0.37

### Visualization

**Figure 6:**
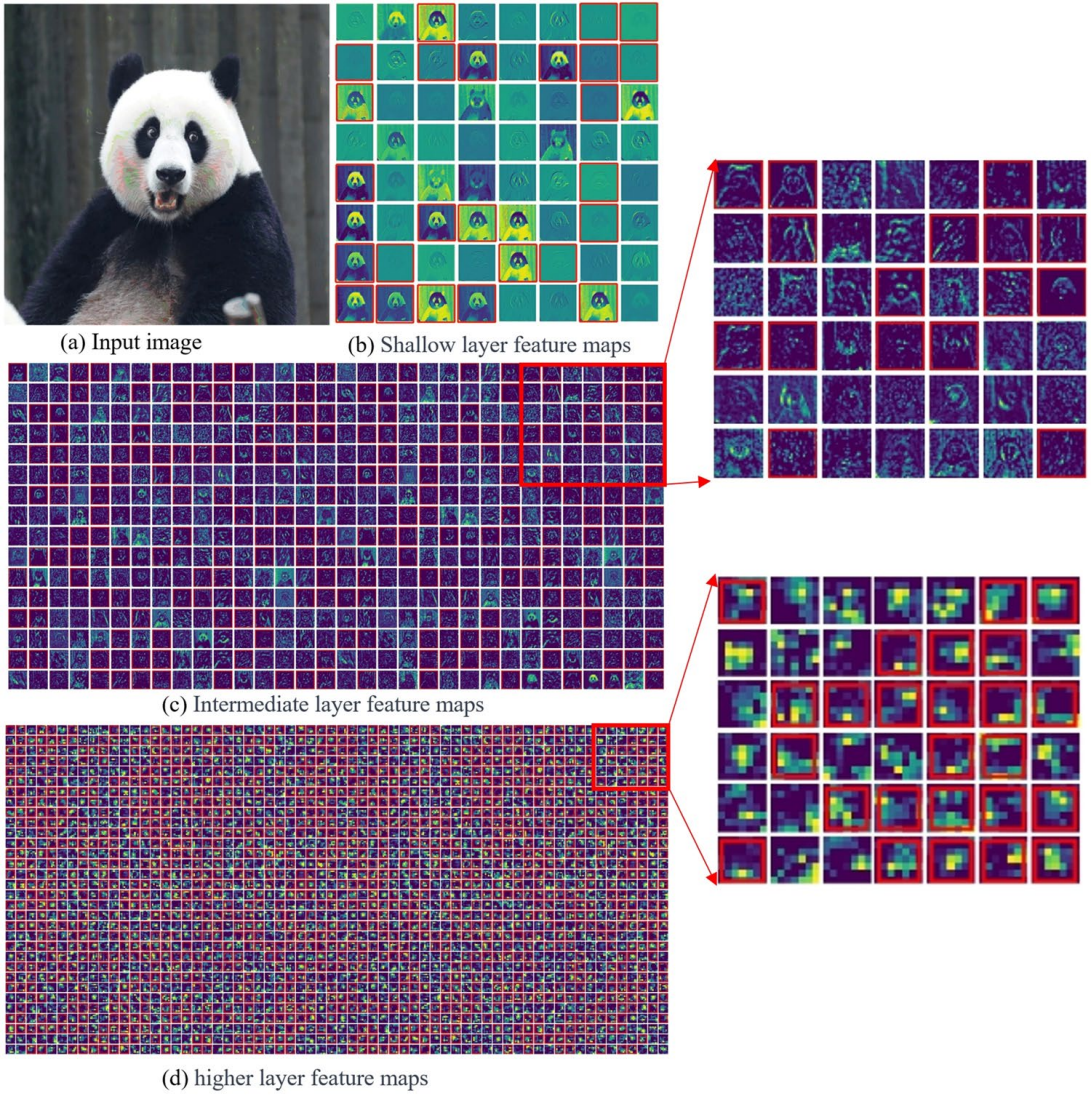
Visualization of different depth convolutional layers of ResNet-50 output feature maps. The feature map in the red box corresponds to the removed filter.

This section presents the application of filter pruning with a 40% pruning rate on the shallow layer (first convolutional layer), intermediate layer (22nd convolutional layer), and deep layer (final layer) of ResNet-50 using CHWP, followed by the visualization of the corresponding output feature maps (Fig. 6). Figure 6a represents the input image, while (b), (c), and (d) depict the output feature maps of various filters in different depth convolutional layers of ResNet-50. Many filters with high similarity or low norms have been removed, as filters pruned in simplifying the network are considered ineffective in extracting valuable features.

## Conclusion

We propose a simple yet effective data-independent method for filter pruning, named CHWP, which aims to facilitate filter pruning. Unlike previous norm-based and relation-based criteria that rank filters solely based on a single type of information, CHWP takes into account the size of filters, dissimilarity between filters, and considers the role of BN layers. This enables CHWP to more efficiently identify the most redundant filters. CHWP, while multifaceted in its considerations, currently has limitations, notably in pruning fully connected layers. Future work will focus on addressing this constraint. Moreover, we aim to integrate CHWP with other acceleration algorithms, including low-precision weights, to advance CNN acceleration further.

## Data Availability

The datasets generated during and analyzed during the current study are available from the corresponding author on reasonable request.
